# Serum thyroid-stimulating hormone and sexual self-schema in domain-specific female sexual function: a cross-sectional study

**DOI:** 10.1093/sexmed/qfag057

**Published:** 2026-07-08

**Authors:** Bayram Özağaç, Melih M Sedef, Mustafa Şanli, Arda B Karaduman

**Affiliations:** Department of Obstetrics and Gynecology, Sungurlu State Hospital, 19300, Corum, Türkiye; Department of Psychiatry, Sungurlu State Hospital, 19300, Corum, Türkiye; Department of Psychiatry, Etlik City Hospital, 06170, Ankara, Türkiye; Department of Obstetrics and Gynecology, Tokat Gaziosmanpasa University Hospital, 60100, Tokat, Türkiye; Department of Obstetrics and Gynecology, Kars Harakani State Hospital, 36200, Kars, Türkiye

**Keywords:** female sexual dysfunction, TSH, thyroid, sexual self-schema, FSFI, cross-sectional study

## Abstract

**Introduction:**

Female sexual dysfunction (FSD) is multifactorial, and thyroid status and sexual self-schema (SSS) may contribute differently across domains of sexual function. The aim of this study was to examine whether serum thyroid-stimulating hormone (TSH) and SSS are independently associated with domain-specific female sexual function.

**Methods:**

This cross-sectional study included 200 sexually active women aged 18–60 years attending a gynecology outpatient clinic between October 2025 and February 2026. Participants completed the Female Sexual Function Index (FSFI) and the SSS Scale. Morning fasting serum TSH was measured with a chemiluminescent immunoassay and clinical threshold was 4 mIU/L. Group comparisons were performed between women with and without FSD defined by a FSFI total score of ≤26.55. Spearman correlations were calculated and adjusted for multiple comparisons using false discovery rate (FDR) correction. Multivariable linear regression models were constructed for FSFI domains and total score, adjusting for age, education, TSH, and SSS. A sensitivity analysis additionally adjusted the arousal model for comorbid hypothyroidism.

**Results:**

Primary outcomes were domain-specific FSFI domains and total score. Female sexual dysfunction was present in 116 of 200 women (58%). Mean TSH did not differ between women with and without FSD. Thyroid-stimulating hormone was negatively correlated with arousal and was not correlated with other domains or total score. Sexual self-schema was associated with desire, arousal, satisfaction, pain, and total score after FDR correction. In multivariable models, higher TSH was independently associated with lower arousal score. Sexual self-schema was independently associated with desire and satisfaction. When TSH was modeled categorically, high TSH remained associated with lower arousal. The TSH-arousal association remained significant after additionally adjusting for comorbid hypothyroidism, which was not independently associated with arousal.

**Discussion:**

In women reporting arousal difficulties, TSH may be clinically relevant even when overall sexual function is not impaired. Strengths of this study include domain-specific modeling, structured exclusion of psychiatric disorders, and evaluation of endocrine and cognitive factors in the same framework. Limitations include cross-sectional design, single TSH measurement without free thyroid hormones or antibodies, reliance on self-report outcomes, and limited representation of overt thyroid disease. Taken together, these findings suggest higher TSH was independently associated with lower sexual arousal, while SSS showed broader associations with desire and satisfaction, supporting a domain-specific biopsychosocial model of female sexual function.

## Introduction

Female sexual dysfunction (FSD) is a highly prevalent and multifactorial condition, with approximately 40%-50% of women experiencing at least one sexual problem during their lifetime.^1^^,^^2^ Female sexual dysfunction is not a unitary disorder but encompasses disturbances in various aspects of sexual response such as desire, arousal, lubrication, orgasm, satisfaction, and pain.^3^ Prevalence rates specific to each domain indicate that difficulties with desire (50.7%) and arousal (48.2%) are the most commonly reported, highlighting the need for domain-specific research.^1^^,^^4^ The causes of FSD are multifaceted, involving a combination of sociodemographic, medical, and psychological factors, such as age, chronic illness, relationship quality, and history of abuse.^1^^,^^5^^,^^6^

Among medical contributors to sexual dysfunction, thyroid disorders have attracted increasing attention. A meta-analysis found that 44.8% of women with thyroid conditions experience FSD, with the prevalence rising to 59.6% in cases of hyperthyroidism.^7^ The relationship between thyroid function and specific sexual aspects is complex and appears domain dependent. Overt hypothyroidism is linked to widespread issues across all areas of the Female Sexual Function Index (FSFI), whereas subclinical hypothyroidism tends to selectively impact arousal and orgasm, without notably increasing the overall risk of FSD.^8^^,^^9^ These results indicate that focusing solely on total FSFI scores might mask important domain-specific physiological patterns, which are crucial for understanding underlying mechanisms and guiding targeted clinical treatments.

Despite emerging evidence linking thyroid dysfunction to sexual difficulties, a critical limitation of prior research is the lack of attention to psychosexual factors. Sexual function is not just a physiological process but is significantly affected by cognitive and affective factors.^10^^,^^11^ Sexual self-schema (SSS), defined as cognitive generalizations about sexual aspects of the self derived from past experiences, plays a central role in shaping sexual information processing and behavior.^12^^,^^13^ Individuals with more positive SSSs tend to experience greater desire, arousal, and satisfaction, along with lower levels of sexual anxiety and avoidance.^12^^,^^14^^,^^15^ Notably, cognitive schema variables explain significant variance in sexual functioning even after controlling for demographic and medical variables.^13^^,^^16^ Therefore, investigations seeking to isolate biological contributions to sexual function must demonstrate that observed endocrine associations are independent of these psychological determinants.

To our knowledge, few studies have examined thyroid function and SSS within the same analytic model to disentangle biological and cognitive correlates of domain-specific sexual functioning. Accordingly, this study examined the associations of serum thyroid-stimulating hormone (TSH) levels and SSS with female sexual function as measured by the FSFI, both at the total score and domain levels. We specifically aimed to determine whether TSH and SSS were independently related to individual FSFI domains and overall sexual functioning. Are serum TSH and SSS independently associated with FSFI arousal and other domains, and does SSS modify the TSH–arousal association?

## Methods

### Study design and participants

This cross-sectional study was conducted at the Gynecology and Obstetrics Outpatient Clinic of Tokat Gaziosmanpasa University Hospital, Tokat, Türkiye, between October 2025 and February 2026. The study protocol was approved by the Institutional Review Board/Ethics Committee of Tokat Gaziosmanpasa University Hospital (Approval No: 26-MOBAEK-090). All participants provided written informed consent prior to enrollment. To reduce selection and information bias, potentially eligible women were assessed consecutively during the study period using predefined inclusion and exclusion criteria. Psychiatric comorbidity was screened by a trained psychiatry specialist using the Structured Clinical Interview for the Diagnostic and Statistical Manual of Mental Disorders, Fifth Edition (DSM-5), and all questionnaire assessments were completed in dedicated sessions held outside routine clinic hours to ensure privacy and standardized data collection.

Women between ages 18-60 years who presented for regular gynecological care were evaluated for eligibility. To be included, participants needed to have an active sexual life, defined as engaging in sexual activity at least once a month over the past 6 months, and be capable of understanding and completing self-administered questionnaires.

Exclusion criteria included current severe psychiatric disorders (eg, schizophrenia, bipolar disorder, major depressive disorder); major endocrine disorders (eg, diabetes mellitus, hyperprolactinemia, Cushing’s syndrome); pregnancy, breastfeeding, or postpartum period within the last 6 months; the use of medications known to impact sexual function, such as antidepressants, antipsychotics, hormonal contraceptives, or hormone replacement therapy; initiation of any medication that might influence thyroid function within the last 3 months; a history of cancer, especially thyroid, breast, or pituitary tumors; history of sexual trauma; severe chronic systemic diseases (eg, renal or hepatic failure); and active alcohol or substance use disorder. Women with known thyroid disease were not excluded if clinically stable and under treatment.

### Data collection and measures

Assessments were conducted by a gynecologist and a psychiatry specialist during dedicated sessions held outside of regular clinic hours to ensure privacy and standardized evaluation.

#### Sociodemographic and clinical data form

A structured form was used to collect information on age, marital status, educational level, occupation, current medications, and medical or psychiatric illnesses.

#### Structured clinical interview for DSM-5

A trained psychiatry specialist administered the Turkish version of the Structured Clinical Interview for DSM-5 (SCID-5) to exclude participants with psychiatric comorbidities. The instrument has demonstrated high inter-rater reliability and has been validated for the Turkish population.^17^

#### Female sexual function index

The FSFI is a self-administered questionnaire consisting of 19 items that assess 6 aspects of female sexual function over the previous 4 weeks: desire, arousal, lubrication, orgasm, satisfaction, and pain.^18^ Scores for each domain are determined by adding the relevant items and then multiplying by specific coefficients for each domain (0.6 for desire; 0.3 for arousal and lubrication; 0.4 for orgasm, satisfaction, and pain), resulting in a total score that can range from 2 to 36. Higher scores indicate better sexual function. A total FSFI score ≤26.55 is considered indicative of FSD, while domain scores below 3.6 suggest dysfunction in that particular area.^19^ The validated Turkish version of the FSFI was used in this study.^20^

#### Sexual self-schema scale

The SSS Scale was utilized to evaluate SSS. Higher overall SSS scores indicate a more positive, open, and romantic sexual self-view, whereas lower (or negative) scores reflect more conservative or embarrassed views and fewer positive sexual attributes.^21^^,^^22^ The Turkish validated 33-item version of the SSS Scale was used. A total SSS score was calculated by summing all 33 items, with higher scores indicating a more positive SSS.^23^ Subscale scores were not used in the primary analyses. The SSS Scale demonstrated excellent internal consistency in the present sample (Cronbach’s α = .93).

#### Biochemical analysis

Venous blood samples were collected between 09:00 and 10:00 AM after a 12-h overnight fast. Serum TSH was measured using fully automated Abbott immunoassay analyzers (Abbott Laboratories, Abbott Park, IL, USA). The laboratory reference range for TSH was 0.4-4.0 mIU/L. TSH was analyzed both as a continuous variable and categorically (<0.4, 0.4-4.0, >4.0 mIU/L).

### Statistical analysis

Sample size was calculated a priori using G^*^Power (Version 3.1.9.7). To detect a correlation coefficient of 0.25 between thyroid parameters and sexual function with α = .05 and 80% power, a minimum of 123 participants was required. To account for incomplete data and enhance reliability, the target sample size increased by approximately 20%.

A post hoc power analysis was conducted using G^*^Power (Version 3.1.9.7) for linear multiple regression (fixed model, *R*^2^ deviation from zero). For the primary arousal regression model, the observed *R*^2^ was 0.080, corresponding to Cohen’s *f*^2^ = 0.087. With α = .05, *N* = 200, and 5 predictors, achieved power was approximately 0.91. However, because this analysis was based on the overall model effect, the study may still have limited power to detect smaller individual predictor effects or interaction effects.

Statistical analyses were performed using IBM SPSS Statistics for Windows, Version 27.0 (IBM Corp., Armonk, NY, USA). The normality of continuous data distribution was assessed using the Kolmogorov–Smirnov test. Continuous variables were reported as mean ± SD or median (interquartile range), and categorical variables as frequencies and percentages. Group comparisons between women with and without FSD (FSFI ≤26.55) were performed using independent samples t-tests or Mann–Whitney U tests for continuous variables and chi-square tests for categorical variables.

Spearman correlation coefficients were calculated to examine associations between TSH levels and FSFI domain scores, FSFI total score, and SSS scores. False discovery rate (FDR) correction was applied to adjust for multiple comparisons across FSFI domains. Multivariable linear regression analyses were conducted to evaluate independent predictors of sexual function. Separate models were constructed for each FSFI domain (desire, arousal, lubrication, orgasm, satisfaction, pain) and for the total FSFI score. Covariates included age, education level, serum TSH, SSS total score. Standardized beta coefficients (β) and *P* values were reported. Model assumptions including normality of residuals and multicollinearity were examined prior to interpretation. Sensitivity analyses were performed for FSFI arousal domain by additionally adjusting regression models for comorbid hypothyroidism to evaluate robustness of primary findings. There were no missing data for the variables included in the primary analyses; therefore, no imputation procedure was required. Only participants with complete questionnaire and laboratory data were included in the final analytic sample.

## Results

### Sample characteristics

A total of 200 women were included in the study. Based on the FSFI cut-off score (≤26.55), 116 participants (58%) were identified as having FSD. The mean age of the sample was 35.0 ± 7.9 years, with no significant age difference between groups (*P* = .971). Marital status and income level did not differ significantly between groups, whereas educational level showed a significant difference (*P* = .008).

Nine participants (4.5%) had a documented diagnosis of hypothyroidism, of whom 8 were receiving thyroid hormone replacement therapy. There was no significant difference in mean serum TSH levels between women with and without FSD (2.90 ± 1.29 vs. 2.56 ± 1.46 mIU/L; *P* = .404) (Table 1).

**Table 1:** Sociodemographic and clinical characteristics of the study sample by female sexual dysfunction status.

	Total (*N* = 200)	FSFI ≤26.55 (*n* = 116)	FSFI >26.55 (*n* = 84)	Test statistic	*P* value
Age, years	35.0 ± 7.9	35.0 ± 8.3	34.9 ± 7.3	−0.036	.971
Marital status (*n* %)				2.860	.414
Single	5 (2.5)	4 (3.4)	1 (1.2)		
Married	189 (94.5)	107 (92.2)	82 (97.6)		
Divorced	5 (2.5)	4 (3.4)	1 (1.2)		
Widowed	1 (0.5)	1 (0.9)	0 (0)		
Education status (*n* %)				11.860	**.008**
Primary school	38 (19.5)	27 (23.3)	11 (13.3)		
Middle school	68(34)	29 (25.0)^a^	39 (47.0)^b^		
High school	59 (29.5)	41 (35.3)^a^	18 (21.7)^b^		
University	34 (17)	19 (16.4)	15 (17.9)		
Income level, *n* (%)				0.310	.856
Low	122 (61)	73 (62.9)	49 (59.0)		
Moderate	59 (29.5)	33 (28.4)	26 (31.3)		
High	18 (9)	10 (8.6)	8 (9.6)		
Any medical comorbidity, *n* (%)	44 (22)	23 (19.8)	21 (25.0)	0.760	.383
Hypothyroidism *n* (%)	9 (4.5)	3 (2.6)	6 (7.1)	2.354	.125
TSH, mIU/L	2.75 ± 1.41	2.90 ± 1.29	2.56 ± 1.46	−0.834	.404
<0.4	1 (0.5)	1 (0.9)	0 (0)	1.033	.597
0.4-4.0	157 (78.5)	89 (78.1)	68 (81.9)		
>4.0	39 (19.5)	24 (21.1)	15 (18.1)		

Data are presented as mean ± SD or *n* (%), as appropriate. Group comparisons were performed using the independent-samples t test for continuous variables and the chi-square test for categorical variables. Different superscript letters indicate significant pairwise differences between groups in post hoc comparisons. Abbreviations: FSFI = Female Sexual Function Index; TSH = thyroid-stimulating hormone.Bold values indicate statistically significant *p* values (*p* < 0.05).

### Comparison of sexual function and sexual self-schema by FSD status

All FSFI domain scores were significantly lower in women classified with FSD compared to those without FSD (all *P* < .001). In terms of SSS, women with FSD had significantly higher total SSS scores (*P* = .033) (Table 2).

**Table 2:** FSFI domain scores and sexual self-schema scores by female sexual dysfunction status.

	Total (*N* = 200)	FSFI ≤ 26.55 *n* = 116	FSFI > 26.55 *n* = 84	*z*	*P* value
FSFI-desire	3.6 (1.2)	3.6 (1.7)	4.2 (1.1)	6.079	<.001
FSFI-arousal	4.2 (1.7)	3.4 (1.5)	5.1 (1.4)	9.844	<.001
FSFI-lubrication	4.5 (1.2)	4.2 (1.1)	5.1 (0.9)	8.699	<.001
FSFI-orgasm	4.4 (1.2)	4.4 (0.7)	5.2 (0.8)	8.904	<.001
FSFI-satisfaction	3.6 (1.9)	3.2 (1.6)	4.2 (1.2)	6.447	<.001
FSFI-pain	5.2 (2.7)	4.8 (2.0)	5.6 (2.4)	3.411	<.001
FSFI-total score	25.8 (4.9)	24 (3.8)	28.7 (2.9)	12.060	<.001
SSS-total score	127 (48)	132.5 (52)	117.5 (40)	−2.134	.033

Data are presented as median (interquartile range). Group comparisons were performed using the Mann–Whitney U test. Abbreviations: FSFI = Female Sexual Function Index; SSS = Sexual Self-Schema Scale.

### Correlations between serum TSH and sexual function

Spearman correlation analyses demonstrated that serum TSH levels were significantly negatively correlated only with the arousal domain of the FSFI (*r* = −0.199, *P* = .035). No significant correlations were observed between TSH and desire, lubrication, orgasm, satisfaction, pain, or total FSFI score. Sexual self-schema total scores were significantly associated with multiple FSFI domains. Higher SSS scores were correlated with lower scores in desire (*r* = −0.287, *P* = .007), arousal (*r* = −0.183, *P* = .016), satisfaction (*r* = −0.300, *P* = .007), pain (*r* = 0.211, *P* = .007), and total sexual function (*r* = −0.116, *P* = .027), whereas associations with lubrication and orgasm were not statistically significant (Table 3).

**Table 3:** Correlations of serum TSH and SSS with FSFI domain scores and total FSFI score.

FSFI Domain	TSH, *r*	95% CI	*P* value^*^	SSS total score, *r*	95% CI	*P* value^*^
Desire	−0.136	−0.274 to 0.008	.200	−**0.287**	−**0.413 to** −**0.151**	**.007**
Arousal	−**0.199**	−**0.333 to** −**0.057**	**.035**	−**0.183**	−**0.318 to** −**0.042**	**.016**
Lubrication	−0.102	−0.243 to 0.042	.266	0.002	−0.141 to 0.145	.980
Orgasm	−0.020	−0.164 to 0.124	.849	−0.135	−0.272 to −0.008	.067
Satisfaction	0.014	−0.130 to 0.157	.849	−**0.300**	−**0.424 to** −**0.164**	**.007**
Pain	0.019	−0.125 to 0.162	.849	**0.211**	−**0.070 to 0.343**	**.007**
Total FSFI score	−0.110	−0.250 to 0.034	.266	−**0.116**	−**0.302 to** −**0.024**	**.027**

Spearman correlation coefficients are shown. ^*^P values were adjusted for multiple comparisons using the FDR method. Abbreviations: CI = confidence interval; FDR = false discovery rate; FSFI = Female Sexual Function Index; SSS = Sexual Self-Schema Scale; TSH = thyroid-stimulating hormone. Bold values indicate statistically significant correlations after false discovery rate correction (adjusted *p* < 0.05).

Serum TSH levels were not significantly correlated with total SSS score (*r* = 0.120, *P* = .094).

### Multivariable linear regression analyses

Multivariable linear regression analyses were conducted to examine independent predictors of FSFI domains, and total scores.

For sexual desire, SSS was independently associated with desire (β = −.242, *P* = .001), whereas TSH was not significant (β = −.096, *P* = .167). Age showed a borderline association (β = .144, *P* = .051). For sexual arousal, higher TSH levels were independently associated with lower arousal (β = −.166, *P* = .019). For sexual satisfaction, SSS was the strongest independent predictor (β = −.274, *P* < .001), whereas TSH was not associated with satisfaction (β = .017, *P* = .803). (Table 4).

**Table 4:** Multivariable linear regression analyses for FSFI desire, arousal, satisfaction, and total FSFI score.

	FSFI-desire		FSFI-arousal		FSFI-satisfaction		FSFI-total score	
	β	*P*	β	*P*	β	*P*	β	*P*
Age	.138	.061	−.052	.486	−.008	.908	−.068	.375
Education (middle vs primary)	.091	.343	.070	.472	**.196**	**.040**	.104	.291
Education (university vs primary)	.002	.984	−.074	.468	−.031	.758	−.068	.515
TSH	−.096	.167	−**.166**	**.019**	.014	.841	−.056	.437
SSS total score	−**.242**	**.001**	−.141	.052	−**.232**	**<.001**	−.085	.247
TSH × SSS (interaction)	**—**	**—**	−.057	.425	**—**	**—**	—	—
*R* ^2^	0.111		0.080		0.116		0.044	
Adjusted *R*^2^	0.087		0.056		0.093		0.019	
*F* (df)	4.55 (5, 191)		3.310 (5, 191)		5.006 (5, 191)		1.752 (5, 191)	
*P* (model)	<.001		.007		<.001		.125	

Standardized beta coefficients (β) are shown for regression coefficients. Primary school was used as the reference category for education level. The TSH × SSS interaction term was tested in an additional arousal model using standardized scores (Δ*R*^2^ = 0.003; *F* change = 0.640; *P* = .425). Abbreviations: FSFI = Female Sexual Function Index; SSS = sexual self-schema; TSH = thyroid-stimulating hormone. Bold values indicate statistically significant *p* values (*p* < 0.05).

To examine whether SSS moderated the association between serum TSH and sexual arousal, an additional interaction model was constructed using standardized TSH and standardized SSS scores. Adding the TSH × SSS interaction term did not significantly improve model fit (ΔR^2^ = 0.003, *F* change = 0.640, *P* = .425). The interaction term was not statistically significant (β = −.057, *P* = .425). In this model, higher TSH remained independently associated with lower FSFI arousal scores (β = −.158, *P* = .027), while SSS was also independently associated with arousal (β = −.148, *P* = .043).

When TSH was modeled categorically (>4 mIU/L), high TSH remained independently associated with lower arousal scores (β = −.178, *P* = .011).

In a sensitivity analysis adjusting for comorbid hypothyroidism, the association between serum TSH and sexual arousal remained statistically significant (β = −.173, *P* = .016). Comorbid hypothyroidism was not independently associated with arousal (*P* = .537), indicating that the observed relationship was not driven by diagnosed thyroid disease.

Additional regression models were conducted for lubrication, orgasm, and pain domains and FSFI total scores. None of these models reached statistical significance. In these models, neither serum TSH levels nor SSS were significant independent predictors. Age and education were also not significantly associated with these domains.

## Discussion

In this cross-sectional study, serum TSH levels were not associated with overall sexual function but showed a modest, independent negative relationship with the arousal domain of the FSFI. This association remained significant after adjustment for age, education, and SSS, and was observed both when TSH was analyzed as a continuous variable and when categorized using the clinical cut-off (>4 mIU/L). In contrast, SSS was independently associated with sexual desire and sexual satisfaction, with a borderline association observed for arousal in multiple models. Notably, TSH levels were not correlated with SSS, indicating that endocrine and cognitive factors contributed independently to sexual functioning in this cohort. These findings suggest a domain-specific pattern rather than a generalized endocrine effect on female sexual function and underscore the importance of assessing both biological and psychological factors in clinical evaluations of FSD.

The selective association between higher TSH levels and reduced arousal scores, regardless of age, education, and SSS, is consistent with meta-analytic findings that suggest subclinical hypothyroidism mainly affects arousal and orgasm rather than causing global deficits across all sexual domains.^7^^,^^8^ A meta-analysis revealed that in subclinical hypothyroidism, only arousal and orgasm scores were significantly reduced compared to euthyroid controls, whereas scores for desire, lubrication, satisfaction, and pain showed no significant differences. In contrast, overt hypothyroidism was associated with impairment across all domains, with lubrication being the most affected.^7^ Additionally, several studies further reported inverse correlations between TSH levels and sexual function scores, particularly in arousal-related domains, indicating a domain-specific rather than generalized endocrine effect.^24–26^ Our findings extend this literature by demonstrating that even within a predominantly euthyroid outpatient sample, higher TSH levels were selectively associated with reduced arousal, supporting a dimensional rather than purely categorical interpretation of thyroid-related sexual effects.

From a physiological perspective, female genital arousal depends on autonomic regulation of pelvic vasodilation and the relaxation of smooth muscle mediated by nitric oxide (NO).^27^ Impaired endothelial NO synthase activity has been implicated in female sexual arousal disorder,^28^ and both overt and subclinical hypothyroidism are associated with endothelial dysfunction and reduced NO availability.^29^^,^^30^ Since genital arousal is highly dependent on endothelial and autonomic integrity, these thyroid-related vascular changes provide a biologically plausible mechanism linking elevated TSH to reduced arousal, even when other sexual domains remain relatively unaffected.^7^^,^^24^^,^^28^ Notably, our sample predominantly consisted of women with TSH levels within or close to the normal range, indicating that even variations within the euthyroid range might have a limited effect on physiological arousal rather than causing a global impact on sexual function. The persistence of this association after controlling for diagnosed hypothyroidism further suggests that the observed effect was not solely driven by clinically overt thyroid disease.

Sexual self-schema emerged as a consistent independent predictor across multiple domains of sexual function, including desire and satisfaction. In contrast to TSH, which demonstrated a domain-specific association limited to arousal, SSS showed broader cross-domain effects, supporting the distinction between endocrine–physiological influences and cognitive–psychological determinants of female sexual function. Previous research indicates that positive and agentic SSSs correlate with increased desire, arousal, and satisfaction, whereas negative schemas are associated with poorer functioning and greater distress.^15^^,^^31–33^

However, we observed a counterintuitive pattern in which higher SSS total scores were associated with lower FSFI scores. This counterintuitive finding may be due to expectation and sampling effects rather than any negative impact of positive SSSs. Women who prioritize sexuality may be more sensitive to subtle difficulties and more willing to label or report them as dysfunction, consistent with evidence that self-perception and sexual values influence symptom appraisal.^34^^,^^35^ In clinical or gynecologic samples, psychological factors such as distress, anxiety, and genital self-image have been shown to lower FSFI scores even when individuals have a generally positive view of their sexual selves.^36^^,^^37^ Therefore, the negative correlations observed in this context are more likely due to increased awareness and the dynamics of seeking help, rather than a genuine adverse effect of a positive SSS on sexual functioning.

The findings for lubrication, orgasm, and pain did not show statistically significance, and neither TSH nor SSS were significant predictors for these domains. This may reflect the multifactorial nature of these specific functions. For example, lubrication is strongly influenced by age, menopausal status, and estrogen levels, with less or no connection to psychosocial characteristics.^31^ Female orgasm depends on coordinated activity of clitoral and pelvic floor structures, neural pathways, and psychological conditions.^38^^,^^39^ Pain during intercourse, known as dyspareunia, is often related to gynecological conditions or pelvic floor dysfunction.^40^ Future research should incorporate domain-specific measures, such as objective lubrication assessments or detailed pain assessments.

This study has several significant strengths. First, the use of domain-specific analytical approach allowed detection of a selective TSH–arousal association that would likely have been obscured by reliance on total FSFI scores alone. Second, the thorough exclusion of psychiatric comorbidities through structured SCID-5 interviews minimized the potential confounding effects of untreated mental health issues. Third, inclusion of SSS enabled simultaneous evaluation of endocrine and psychological contributions within the same framework. Fourth, FDR correction was applied in correlation analyses to control for Type I error. Finally, a priori sample size calculation ensured adequate statistical power to detect the anticipated associations.

It is important to recognize several limitations. The cross-sectional nature of study prevents establishing causality, and the direction of the TSH–arousal association cannot be determined. TSH was only measured at a single time point, which does not capture pulsatile secretion or long-term thyroid status, and free T3, free T4, and thyroid autoantibodies were not assessed, limiting mechanistic interpretation. The sample was drawn from a single tertiary gynecology clinic and mainly included euthyroid women (mean TSH 2.75 ± 1.41 μIU/mL), which enhances internal homogeneity but limits generalizability and the ability to assess the full range of thyroid disorders. A small proportion of participants had diagnosed hypothyroidism and/or were receiving thyroid hormone therapy. The low number precluded subgroup analyses and may limit interpretation across the full spectrum of thyroid dysfunction. The inclusion of a small number of women with treated hypothyroidism represents a clinically heterogeneous subgroup. Although sensitivity analyses adjusting for comorbid hypothyroidism suggested that the TSH–arousal association was not explained solely by diagnosed thyroid disease, the small number of affected participants precluded stratified analyses by thyroid disease and treatment characteristics. Sexual function and SSS were assessed using self-report instruments, which may introduce bias and sociocultural effects, and objective physiological measures of genital arousal were not included. Residual confounding cannot be excluded. Several factors known to influence female sexual function were not measured in detail and/or were not included in the multivariable models, including body mass index, menopausal status, relationship quality, partner factors, subclinical depressive/anxiety symptoms, and hormonal contraception. Finally, the unexpected direction of some bivariate SSS–FSFI correlations suggests the need for replication in community-based samples.

Despite these limitations, the results have important clinical implications. For women experiencing unexplained arousal difficulties, evaluation of thyroid function, including serum TSH, may be reasonable even in the absence of overt hypothyroidism. On the other hand, for women with elevated TSH levels who also report arousal problems, improving thyroid status could improve sexual functioning. Moreover, the consistent association between SSS and multiple domains of sexual function highlights the clinical importance of addressing cognitive and self-referential processes. Therefore, psychotherapeutic approaches that focus on changing negative sexual self-perceptions might serve as a valuable supplementary method in treating FSD, regardless of endocrine status.

## Conclusions

In this study, higher serum TSH levels were independently associated with lower sexual arousal, both as a continuous variable and when categorized using the >4 mIU/L clinical threshold. No significant associations were observed for other domains of sexual function. Sexual self-schema demonstrated broader associations, particularly with desire and satisfaction. These findings support the value of domain-specific analyses in FSD research and reinforce a biopsychosocial framework in which endocrine factors may selectively influence physiological readiness, whereas cognitive self-representations exert wider effects on motivational and evaluative aspects of sexual experience. Prospective and interventional studies are required to clarify causality and to determine whether optimization of thyroid function translates into measurable improvements in sexual arousal.
